# Polymeric Materials for Conversion of Electromagnetic Waves from the Sun to Electric Power

**DOI:** 10.3390/polym10030307

**Published:** 2018-03-12

**Authors:** SK Manirul Haque, Jorge Alfredo Ardila-Rey, Yunusa Umar, Habibur Rahman, Abdullahi Abubakar Mas’ud, Firdaus Muhammad-Sukki, Ricardo Albarracín

**Affiliations:** 1 Department of Chemical and Process Engineering Technology, Jubail Industrial College, P.O. Box 10099, Jubail 31961, Saudi Arabia; Haque_m@jic.edu.sa (S.M.H.); Umar_y@jic.edu.sa (Y.U.); 2 Department of Electrical Engineering, Universidad Técnica Federico Santa María, Santiago de Chile 8940000, Chile; jorge.ardila@usm.cl; 3 Department of General Studies, Jubail Industrial College, P.O. Box 10099, Jubail 31961, Saudi Arabia; habibur_r@jic.edu.sa; 4 Department of Electrical and Electronics Engineering, Jubail Industrial College, P.O. Box 10099, Jubail 319261, Saudi Arabia; 5 School of Engineering, Robert Gordon University, Garthdee Road, Aberdeen AB10 7QB, Scotland, UK; f.b.muhammad-sukki@rgu.ac.uk; 6 Departamento de Ingeniería Eléctrica, Electrónica, Automática y Física Aplicada, Escuela Técnica Superior de Ingeniería y Diseño Industrial, Universidad Politécnica de Madrid, Ronda de Valencia 3, 28012 Madrid, Spain; ricardo.albarracin@upm.es

**Keywords:** polymeric materials, photovoltaic energy, electric power

## Abstract

Solar photoelectric energy converted into electricity requires large surface areas with incident light and flexible materials to capture these light emissions. Currently, sunlight rays are converted to electrical energy using silicon polymeric material with efficiency up to 22%. The majority of the energy is lost during conversion due to an energy gap between sunlight photons and polymer energy transformation. This energy conversion also depends on the morphology of present polymeric materials. Therefore, it is very important to construct mechanisms of highest energy occupied molecular orbitals (HOMO)s and the lowest energy unoccupied molecular orbitals (LUMO)s to increase the efficiency of conversion. The organic and inorganic solar cells used as dyes can absorb more photons from sunlight and the energy gap will be less for better conversion of energy to electricity than the conventional solar cells. This paper provides an up-to-date review on the performance, characterization, and reliability of different composite polymeric materials for energy conversion. Specific attention has been given to organic solar cells because of their several advantages over others, such as their low-energy payback time, conversion efficiency and greenhouse emissions. Finally, this paper provides the recent progress on the application of both organic and inorganic solar cells for electric power generations together with several challenges that are currently faced.

## 1. Introduction

The growth of a country directly depends on the available energy sources. Among all sources, solar energy is one of the most important sustainable energy sources for the future development. It is well known that huge amounts of radiation from the sun reach our ecosystem mainly in the form of energy. After reflection and absorption of the sun light in the atmosphere, approximately 100,000 million MW strikes on the surface and can be converted into electrical energy for the benefit of human progress [1]. According to the International Energy Agency (IEA), this is almost 6000-fold more than the current primary energy consumption worldwide [2]. Therefore, solar energy can be used as an alternative for petroleum and nuclear energy sources and as an important component without affecting the greenhouse and the ecosystem.

The two major solar energy technologies are the solar photovoltaic (PV) and solar thermal systems [3]. Solar PV produces electricity from the sun electronically through a mechanism in a certain type of semiconductor material. The sunrays initiate free electrons from these materials, so that they travel in an electrical circuit to power electrical systems. The PV panels can be mounted on the ground, standing structures or on rooftops. On the other hand, the solar thermal technology produces heat for heating fluids and for operating solar refrigeration systems [4]. Certainly, the solar energy will reduce the environmental problems and increase the economic growth. In the early of nineteenth century, an Italian chemist, Giacomo Luigi Ciamician said the solar energy will be used as important energy resource in the future, and argued that the radiant energy will be a better energy source than the fossil energy [5]. 

The advantages of solar energy will constrain us to think about how to convert the solar energy into electrical energy. The most popular technique is to use solar cells to directly change solar energy to electricity while minimizing the losses. For the conversion of energy, different principles and materials were proposed with practical methodology and still 100% efficiency has not been achieved. Nowadays, scientists are working towards achieving optimum energy conversion and for that purpose, it is crucial to develop or modify the principle as well as equipment’s technology. In the present scenario, the solar energy can be transformed by utilizing photo-catalysts and solar cells. However, one issue with photo-catalyst is the generation of hydrogen fuel after splitting water. 

**Figure 1:**
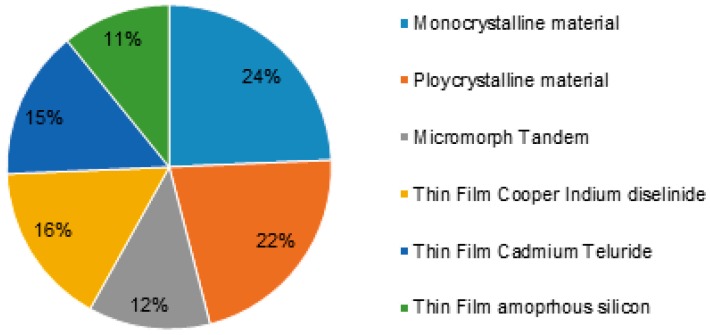
Efficiency of material used in energy conversion.

**Figure 2:**
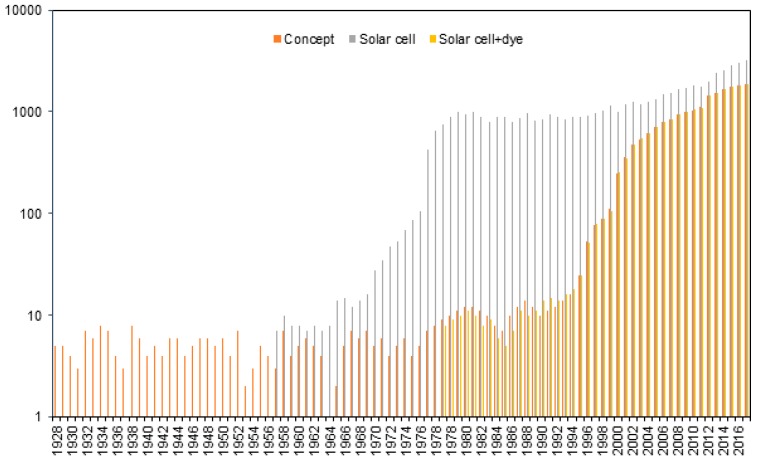
A statistical graph of the number of publications related to the concept of solar cell, solar cell, and dye solar cell. Data were obtained from the Scopus database and Sci Finder.

The studies revealed different types of solar cells that can be used for energy conversion, namely organic polymer [6], silicon [7,8,9], dyes [10,11,12,13], hybrid material [14,15] and copper indium gallium selenide [16,17] solar cell. Figure 1 provides the efficiency of the polymeric materials used in energy conversion. The history of solar cells development gave the clear picture that ‘dye’ is one of the keywords related to solar cells as the researchers found from chemical abstracts. Figure 2 provides the number of documents related to concept of solar cells, solar cells and dye-sensitized solar cell is indicated by purple bar. The solar cell was first reported in 1958 and the PV technology continuously being developed. The solar cells prepared using silicon is highly significant due to its mature fabrication methodology and high-efficiency to conversion reaching approximately 22% [18]. 

The polymer scientists proposed a low-cost conversion technique involving thin film polymer-based organic PV cell. The high-speed conversion reel-to-reel (R2R) processing was developed by introducing coating and printing [19,20,21,22,23,24]. The process consists of plastic material and microcrystalline layer. The rate of deposition of layer was a significant factor for film production. The transistor was made with thin film to create the circuit that can provide regular design of large substrates. The solar cells are depending on exchanging of electron between donor and acceptor. The requirements of compound to be acceptors are that they have the affinity towards electron and can easily transport charge. Carbon-based compound fullerene is used in the form of a large spherical molecule. The composites of polymer and fullerene are dominant due to their capability to convert 8–10% energy from the sun [25,26,27]. The morphology of fullerenes in terms of size (donor and acceptor phase) for bending physically is not easy to control with ordering that can significantly increase the cost of the process. The advancement of photon capturing, charge generation and transport through band gap during sunlight radiation are the key factors for the success of organic polymeric solar cells. Some studies revealed that the charge mobility and separation depend on the bulky structure of the polymer. Absorption of photon results in an electron from molecule being excited to a higher energy state and loses electron to the adjacent molecule [25,26,27,28,29,30,31,32]. The efficiency of organic polymeric material for conversion of energy depends on some important factors [28,29]. These include the improvement of electron capturing ability, generation of charge, diffusion and collection of electrodes and charge transport between donor and acceptor electron.

The fundamental requirement for electron capturing is polymer material’s excitation energy must be equal to incident photon energy. The energy gap is based on the materials, design, and synthesis. The scientists are continuously searching for different polymer materials to form composites. The morphology of active layer and electrical properties of composites are investigated for the improvement. Generally, PV cell used indium and tin oxide (ITO) elements as transparent electrode [19,20,21,22,23,24,33]. However, the disadvantage of applying ITO was due to its mechanical property—its flexible nature could cause crack, lead to degradation, and decrease the performance of the PV cell. The conductivity of poly (3,4-ethylenedioxythio-phene) and poly (4-styrenesulphonate) (PEDOT:PSS) are high and could be used as replacement for ITO [34,35,36,37,38,39]. This polymer does not have the ability to provide greater efficiency with large surface area. Nevertheless, their properties can be improved by incorporating metal on it [40,41,42]. The cost-effective organic photovoltaic cell (OPV) was made by printing metal grid. The silver metal has a significant role as the grid and is printed as the last layer of the OPV solar cell [22]. However, in case of inkjet printer, the grid was first printed as the layer and works as the anode in the OPV cell [43]. 

Inorganic material can be employed as an alternative energy conversion source, but it required high-temperature with a vacuum and costly raw materials. Not only that, the process also needed huge amount of money and loses large quantity of energy which restricted the application of the method for the development [44,45,46]. In comparison with organic polymeric R2R PV solar cell is efficient to change materials in the morphology. It has the highest energy occupied molecular orbitals (HOMO, electron donor), lowest energy occupied molecular orbitals (LUMO, electron acceptor) and energy difference of valence band with conduction band (energy gap) with low cost method [28,29,30,31,32] gave opportunity to apply the method for the future. In addition, the semiconductors made up from organic polymeric material present larger optical absorption coefficient than the inorganic material. 

The aim of this review article is to evaluate the significance of solar energy and the performance of different composite polymer materials for solar energy conversion. The article will provide the reader with comprehensive overview of different polymers and polymeric composite that are successfully used on solar energy conversion. The synthesis route and characterization of the polymer materials used for such application will also be presented. In addition, the article reviews the productivity, reliability and compares different solar technologies. Therefore, basic principle and theory about synthesis and electrical properties are discussed with different energy conversion pathway as well as technical challenges. The reference in review will provide the reader with more detailed information about transformation of solar energy to electrical energy.

### 1.1. Polymeric Material

The conjugated polymers can be used in electronics and photovoltaic cells and it showed excellent results with low cost [31]. Polymer-based solar cells have the efficiency to convert power 5–10% in recent reports [25,26,27,47,48,49,50,51]. Screen printing, inkjet printing and spray deposition method easily applicable at lower temperature for organics because materials are soluble in nature. Roll to roll high throughput processing are required the above technique that lowering the cost related to current grid electricity with polymer-based PV solar cell. We discuss current technical challenge as well as basic material needed to increase the efficiency of solar cells. This review article can be a useful guidance for researchers in the field of solar energy conversion and to develop better material systems of PV cell that have efficiency more than 10%.

**Figure 3:**
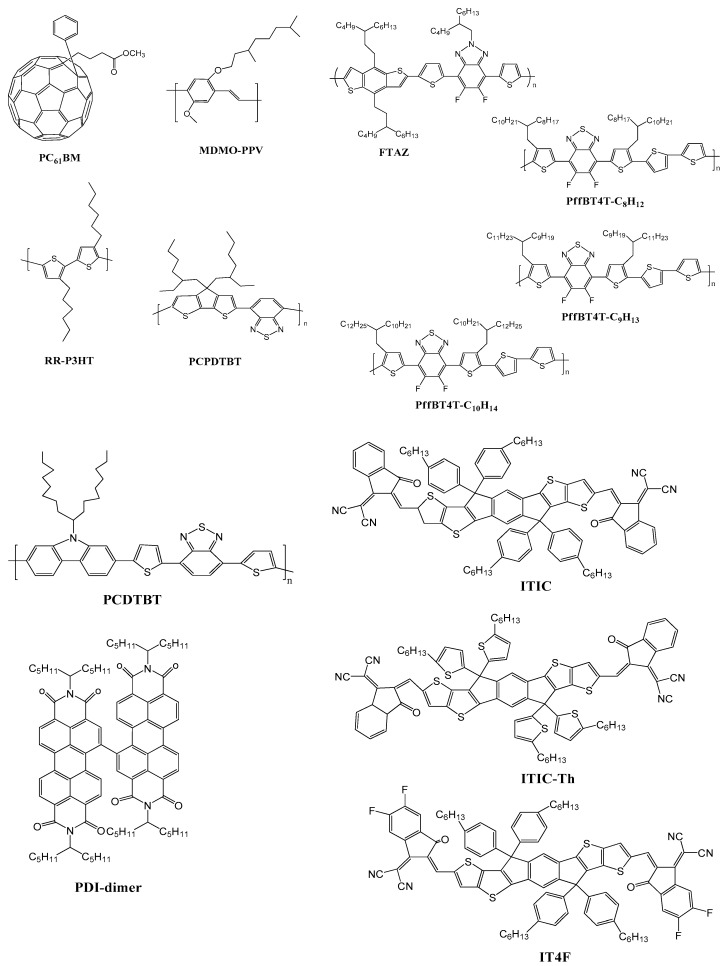

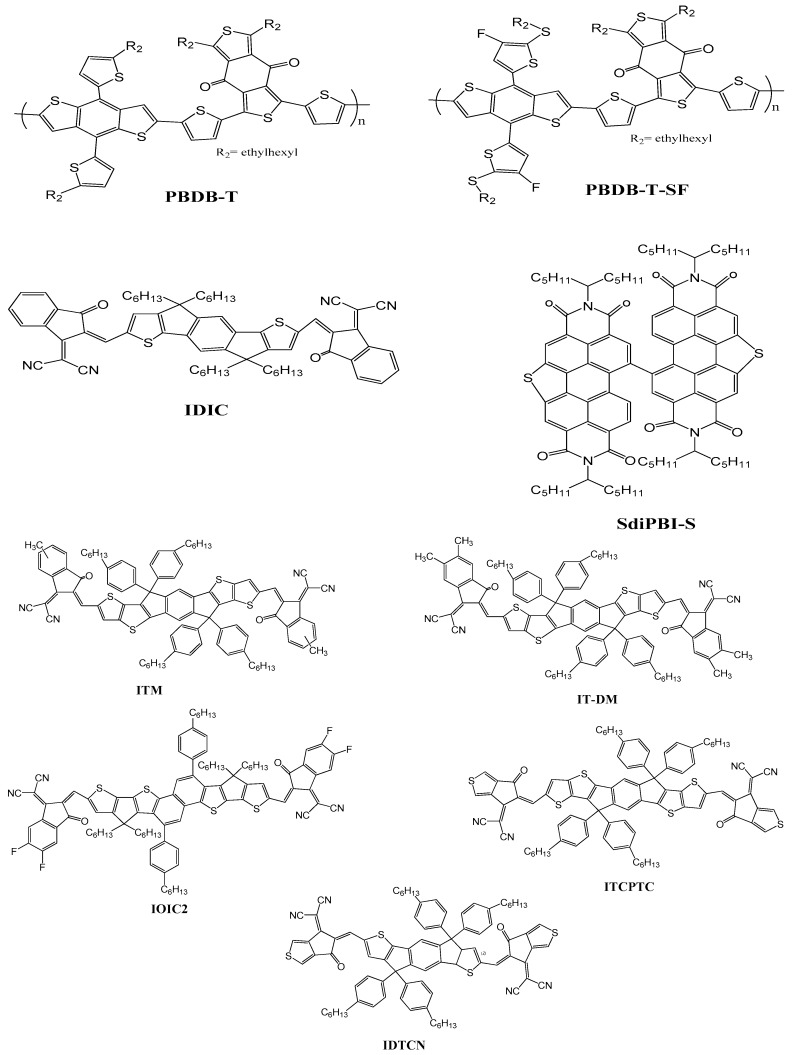
Common materials used in polymer photovoltaics. **PCBM:** (6,6)-phenyl-C61-butyric acid methyl ester; **MDMO-PPV:** poly(2-methoxy-5-(3’,7’-dimethyloctyloxy)-1,4-phenylene-vinylene); **RR-P3HT:** regioregular poly(3-hexylthiophene); **PCPDTBT:** poly[2,6-(4,4-bis-(2-ethylhexyl)-4H-cyclopenta[2,1-b;3,4-b]-dithiophene)-alt-4,7-(2,1,3-benzothiadiazole)]; **PCDTBT:** Poly[N-9′-heptadecanyl-2,7-carbazole-alt-5,5-(4′,7′-di-2-thienyl-2′,1′,3′-benzothiadiazole)]; **PffBT4T-C9C13:** Poly[(5,6-difluoro-2,1,3-benzothiadiazol-4,7-diyl)-alt-(3,3-di(2-nonyltridecyl)-2,20;5,2;5,2-quaterthiophen-5,5-diyl)]; **ITIC:** 3,9-bis(2-methylene-(3-(1,1-dicyanomethylene)-indanone))-5,5,11,11-tetrakis(4-hexylphenyl)-dithieno[2,3-d:2′,3′-d′]-s-indaceno[1,2-b:5,6-b′]dithiophene; **ITIC-Th:** 3,9-bis(2-methylene-(3-(1,1-dicyanomethylene)-indanone))-5,5,11,11-tetrakis(5-hexylthienyl)-dithieno[2,3-d:2′,3′-d′]-s-indaceno[1,2-b:5,6-b′]dithiophene; **IT-4F:** 3,9-bis(2-methylene-((3-(1,1-dicyanomethylene)-6,7-difluoro)-indanone))-5,5,11,11-tetrakis(4-hexylphenyl)-dithieno[2,3-d:2′,3′-d′]-s-indaceno[1,2-b:5,6-b′]dithiophene; **PBDBT:** Poly[(2,6-(4,8-bis(5-(2-ethylhexyl)thiophen-2-yl)-benzo[1,2-b:4,5-b′]dithiophene))-alt-(5,5-(1′,3′-di-2-thienyl-5′,7′-bis(2-ethylhexyl)benzo[1′,2′-c:4′,5′-c′]dithiophene-4,8-dione)]; **IDIC:** 2,2′-((2Z,2′Z)-((4,4,9,9-tetrahexyl-4,9-dihydro-s-indaceno[1,2-b:5,6-b′]dithiophene-2,7-diyl)bis(methanylylidene))bis(3-oxo-2,3-dihydro-1H-indene-2,1-diylidene))dimalononitrile; **FTAZ:** Fluorine substituted benzotriazole; **IRCPTC:** (3,9-bis(2-methylene-(3-(1,1-dicyanomethylene)-cyclopentane-1,3-dione-[c]thiophen))-5,5,11,11-tetrakis(4-hexylphenyl)-dithieno[2,3-d:20,30-d0]-s-indaceno[1,2-b:5,6-b0]dithiophene); **IT-M:** 3,9-bis(2-methylene-((3-(1,1-dicyanomethylene)-6/7-methyl)-indanone))-5,5,11,11-tetrakis(4-hexylphenyl)-dithieno[2,3-d:2′,3′-d′]-s-indaceno[1,2-b:5,6 b′]dithiophene; **IDTCN:** 4,4,9,9-tetrakis(4-hexylphenyl)-4,9-dihydro-s-indaceno[1,2-b]thiophene-alt-[5,6-d]thieno[3,2-b]thiophene)-2-(5/6-methyl-3-oxo-2,3-dihydro-1H-inden-1 ylidene)malononitrile; **IOIC2:** 2,2′-((2Z,2′Z)-(5,10-dihexylnaphtho[1,2-*b*:5,6-*b*′]di(4,4-bis(4-hexylphenyl)-4H-cyclopenta[2,1-*b*:3,4-*b*′]dithiophene-2,7-diyl)bis(5,6-difluoro-3-(dicyanomethylene)-2-methylene-indan-1-one) [75,76,77].

The common materials are used in polymer photovoltaics are PCBM: (6,6)-phenyl-C_61_-butyric acid methyl ester; MDMO-PPV: poly(2-methoxy-5-(3′,7′-dimethyloctyloxy)-1,4-phenylene-vinylene); RR-P3HT: regioregular poly(3-hexylthiophene); PCPDTBT: poly[2,6-(4,4-bis-(2-ethylhexyl)-4H-cyclopenta[2,1-b;3,4-b]-dithiophene)-alt-4,7-(2,1,3-benzothiadiazole)] (Figure 3). All conjugated molecules are highly polarizable due to π-systems so they are electronically active, and easily hybridized the orbitals depending on p atomic orbitals. The π−π* optical transitions are strong enough to tune synthetically by molecular design and typically fall in the visible. The electron hole pair was established when electron excited from HOMO to LUMO during photon absorption that relaxes with binding energy called excitation [52,53,54]. Organic semiconductor has larger binding energy than inorganic due to electron and hole localization and low dielectric constant, which increase the Coulomb attraction between them. Planar heterojunction can be promoted as excitation separation between transparent conductor such as fluorinated tin oxide, indium–tin-oxide coated with poly(styrene sulfanate) and poly(3,4-ethylenedioxythiophene) with reflecting aluminum (Al) or silver (Ag) metal [55]. The geminate pair is formed by internal field after dissociation [56,57,58,59,60]. The most successful device for PV cell is bulk heterojunction (BHJ) because all excitons produced near to heterojunction. The BHJs are formed by diffusion of polymer and accept electron from solvent such as fullerenes [47,48,49,50,51,61,62,63,64,65,66,67,68,69,70] and polymers [71,72,73,74]. TiO_2_ or ZnO are used in organic hybrid cells because all are transparent metal oxide with high electron mobility and easy for processing. 

The important parameter for developing efficient solar cells with polymer materials is to confirm that exciton diffusion length must be more than the lengths of the materials (two) are intermixed. So easily formed exaction reach to the interface with electron acceptor during charge transfer. Solvent and blending ratio are playing a vital role for the performance of solar cell [63,78,79]. Nanostructured TiO_2_ is extremely powerful approach for developing photo voltaic cell because it is nontoxic and abundant for dye sensitized solar cell [80,81]. The performance of the above said solar cell can be improved by incorporating dyes, phosphoric acid group and carboxylic acid group on the surface of nanostructured oxide that increase the charge transfer capability as well as polymer wetting [82,83]. Nowadays, the perovskite solar cells can convert power up to 22.1%. To achieve this target, two important hole-transporting materials 2,20,7,70-tetrakis(*N*,*N*-di-*p*-methoxyphenylamine)-9,90-spirobifluorene and poly-triarylamine are playing significant role [84].

The non-fullerene acceptors (NFAs) are moderately weaker to gain electron compare to fullerene but it has better blend morphology and efficient capability to transfer charge without back transfer with respect to donor material. The materials have greater solubility in all environmentally friendly solvent that give the advantages to synthesize the material easily. The NFAs material frontier energy levels are favorable to the donor material due to its optical absorptivity and structural flexibility. The core unit of NFAs is perylene diimide (PDI). The most commonly used NFAs are ITIC, ITIC-Th, SdiPBI-S, IT-4F, IDIC, ITM, IT-DM, ITCPTC, IDTCN, IOIC2 and donor PBDBT, PBDBTSF, FTAZ (Figure 3) [85,86,87,88,89,90,91,92,93,94,95,96,97,98,99,100,101,102,103,104].

### 1.2. Solar Cells and Solar Power

**Figure 4:**
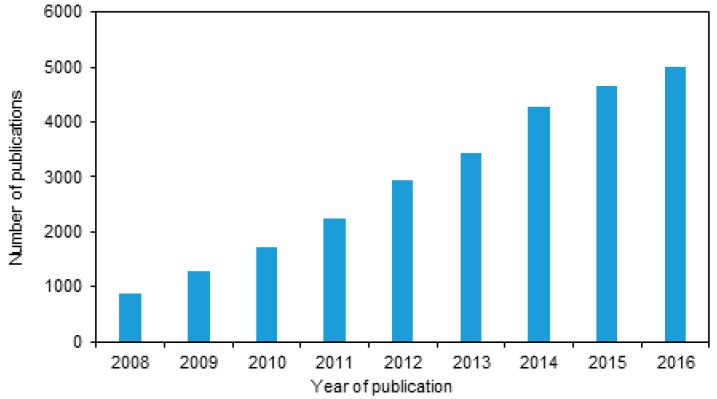
Number of academic publications in organic solar cells up to 2016 [ISI web of knowledge].

In recent years, an ever-growing energy demand has been consolidated through the world. To supply this demand, several renewable energy (RE) sources have been implemented, such as wind, solar, biomass, fuel cells and geothermal [105]. Among these, a great interest has been developed in solar power, because it is abundant, non-polluting and in-expensive [106]. Planet Earth receives $1.75\times 10^{17}\;W$ of solar energy per year; this is enough to satisfy the world annual energy demand in less than an hour [107]. The main technology to harness solar power is solar cells. Among the latest type of solar cell that can be used for this purpose, include the organic solar cells made from organic materials and polymers [108]. The investment in solar cells is high in the present [109], however the cost related to solar power are expected to fall in the next few years [110]. For this reason, several new studies have been developed in the recent years aiming for better efficiency [111]. Examples of this, are dye sensitized solar cells [81,112,113] composed in its core by a wide-bandgap oxide semiconductor, where bandgaps of $TiO_{2}$ [114,115] and ZnO [116,117] have been studied in depth. Other examples are organic solar cells [118,119], composed of an active layer made of a donor and acceptor [109], which can be layered to make a “heterojunction” [120,121]. This promotes the correct splitting and dissociation of the exciton, thus increasing efficiency [111]. In this document, a brief overview of the solar cell history, types, and strategies to improve its efficiency shall be discussed. Table 1 summarizes the different advances and discoveries associated with the solar cells in the last decades [107]. Furthermore, specifically the number of publications regarding the organic solar cells have rose over the years as shown in Figure 4.

### 1.3. Organic and Inorganic Material 

Depending on the type of material used, solar cells can be categorized into the following [111]:

1. Polycrystalline inorganic cells: made of inorganic materials such as $\mathrm{Cu}\left(\mathrm{In},\;\mathrm{Ga}\right){\left(S,\;\mathrm{Se}\right)}_{2}$, CIGSSE, ${\mathrm{Cu}}_{2}\mathrm{ZnSn}{\left(S,\;\mathrm{Se}\right)}_{4}$ and CZTSSe, using gallium (Ga) and Sulfur (S) in ${\mathrm{Cu}}_{2}{\mathrm{InSe}}_{2}\;\left(\mathrm{CISe}\right)$, creates CIGSSe achieving efficiency of 21.7% [122]. Replacing In for Zn and Ga for Sn, creates ${\mathrm{Cu}}_{2}\mathrm{ZNSN}{\left(S,\;\mathrm{Se}\right)}_{4}$ or CZTSSe decreasing manufacture costs and achieving an efficiency of 13% [27].
2. Amorphous Si (a−Si): amorphous solar cells are made of silicon through chemical vapor deposition [123], the conductivity of this cell can be controlled through incorporating phosphine or diborane gas during deposition, preventing efficiency loss [124]. A variation of this cell is made by incorporating hydrogen, generating hydrogenated amorphous silicon (a−Si H), which compared to the a−Si exhibits a better absorption coefficient [105], the highest efficiency recorded for this cell is 13% [116]. Amorphous solar cells/alloys possesses great absorption coefficients that resemble the direct bandgap semiconductor [125].
3. Organic photovoltaics: composed of organic materials by solution-based process [117], due a short diffusion length this type of cell, lead to efficiency near 100%. This issue was fixed by incorporating a bulk distributed interface [126]. Besides achieving efficiency of 12% [127], this type of cell, leads as a candidate for the cost effective photovoltaics [128]. Organic photovoltaics (PVs) differ considerably from the inorganic PV devices in their mode of operation. They can be fabricated by printing, evaporation of the vacuum and applying proper coating techniques [125]. This process provides the potential for more economical mass-producible PV systems.
4. Organic-inorganic halide perovskite: The first Organic-inorganic halide (also known as “perovskite”) is the dye-sensitized solar cell (DSSC or Graetzel cell), made by Graetzel as an extension of the bulk distributed interface [129]. This cell divides the process of absorption, charge transportation and collection in the photovoltaic device [129]. The first implementation of this cell achieved an efficiency of 3.8% [130], in the year 2012 this kind of cell was improved to 9.7% [131]. In the recent years, perovskite has become one of the main research field in high-optical absorption, long-diffusion length and low-recombination rate, which leads to a higher power conversion efficiency [118].

However, it is necessary to emphasize that inorganic solar cells dominate the overall market, but their main disadvantage is being rigid and heavy. For lightweight installations, organic solar cells can be built to be flexible [122], semitransparent for buildings and vehicles [27], and can be fabricated at low-cost by avoiding high-temperature and vacuum process. On the other hand, organic semiconductors enable to manufacture solar cells with thinner films, because of its high-absorption coefficient [123]. The main challenge for the organic solar cells is to achieve high-efficiency, while keeping a long-term stability. Materials implemented in the manufacture of solar cells are crucial for improving the radiation resistance [116,117], of its components, semiconductors and integrated circuits [126,127]. Likewise, organic solar cells can be classified in 3 types, due its structure [107]:

1. Single layered: are the first generation of organic solar cells. They were made from organic layers [118], between metal electrodes [120]. This type of structure has a low-efficiency, due to the generation of low-charge lately this has been improved to an efficiency of 5.9% [132].
2. Bilayer or multilayer structures: are organics layers overlapping [31] first the donor type “p” and then the acceptor type “n”, by this process, are created excitons which are the a electron state, where it gets excited out of its valence band to the conduction band. These excitons increase the energy generation by displacing from donor to acceptor. In recent years, different materials have been studied for donor and acceptor [133,134].
3. Bulk heterojunction structures [135,136]: have a mix of donor and acceptor in the bulk, which improves the interfacial area preventing exciton diffusion [137].

## 2. Synthesis and Characteristics of Polymer Matrix Composite

### 2.1. Phenyl-C61-Butyric Acid Methyl Ester (PCBM) and Poly (3-Hexyl Thiophene) (P3HT)

The anionic surfactants have important role for the synthesis of PCBM and P3HT composites nanoparticles. The positively charged sodium dodecyl sulfate (SDS) surfactant was dissolved in water and 1-propanol followed by mini emulsion technique. Chloroform used as solvent for P3HT. Probe sonication method was developed for the addition of polymer and SDS with heating at 65 °C. The different sizes of nanoparticles are formed by changing weight percent of polymer and various concentration of SDS. The NPs with different size distributions were fabricated with the polymer solutions of different weight percent and at various SDS concentrations. Same procedure was followed for PCBM nanoparticles. In the case of positively charged hexadecyltrimethylammonium bromide (CTAB) was used as surfactant. The PCBM (positive) and P3HT (negative) nanoparticles were mixed heterogeneously by sonication. The desired thickness of layered thin film was achieved by repeating the process several times.

The particle size of the nanoparticles cannot be changed when pseudo steady state reached after sonication but initially the size is increased with the mechanical agitation. The poly diversity is increased with constant fusion and fission forces to reach the steady state. The surfactant molecules are not fully used to cover droplets surfaces in the continuous phase so chiroform are evaporated and trapped organic solvent results to form stable nanoparticles. The thickness of P3HT nanoparticles (negatively charged) are studied using UV–Visible spectroscopy. The spectra of P3HT nanoparticles, P3HT thin film, thin film synthesizes with polymer nanoparticles and P3HT polymer solution are constructed [138]. The studies demonstrated P3HT nanoparticles spectra have slightly shifted in the chloroform with P3HT polymer because of interchain interaction. The micelle size will increase with the increase of SDS concentration and as a result, zeta potential is decreases that increase the hydrodynamic radius. 

The nanoparticles of P3HT and PCBM with opposite charge are attached with each other closely by electrostatic attraction can be proved by atomic force microscope (AFM) in tapping mode. The phase difference with reference drive signal is more with the region of soft and elastic material than the harder material [139]. The softer P3HT nanoparticles are higher phase and harder PCBM nanoparticles are lower phase in AFM image [140]. Photovoltaic response can be measured after making solar cell with P3HT and PCBM single layered composite nanoparticles. PCBM and conjugated polymer nanoparticles are used to produce bulk heterojunction solar cells. 

### 2.2. Poly(Ethylene-3,4-Dioxythiophene) (PEDOT) and Poly (Styrene Sulfonic Acid) (PSS)

The stable graphene suspension prepared in isopropyl alcohol with two hours of sonication and large sheets are breaking into small to from mixture. The color of filtrate mixture is black after 48 h [141]. The pristine PEDOT:PSS aqueous solution is prepared with PEDOT (0.5%) and PSS (0.8%) in water. Then the PEDOT:PSS solution are mixed with graphene filtrate (2:1, *v*/*v*) [142,143] in the presence of additives or surfactants with continuous stirring for 3 h to form thin film and later on using coating equipment to spray on it. During this process, droplets formed by ultrasonic nozzle are moved towards the substrate by air. The droplets are needed to control because it will splash on the substrate as well as scatter, hence fix the pressure by monitoring the vibration frequency and flow rate of the PEDOT: PSS solution. The substrate temperature during spraying process was maintained by putting transducers into water bath at 80 °C [144].

**Figure 5:**
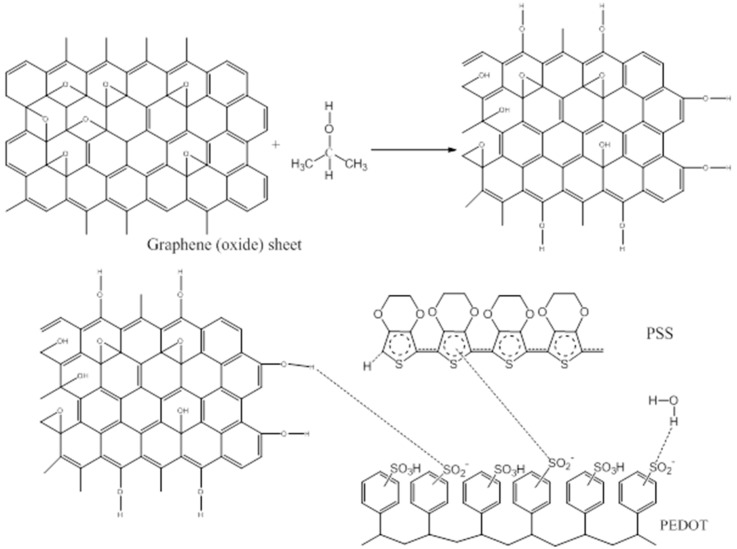
Proposed synthesis route and chemical interactions between graphene and PEDOT:PSS in IPA and water.

Isopropyl alcohol is better for dispersion of graphene than dimethyl formamide and water to form black stable suspension. The confocal laser scanning microscope and AFM studies on filtrate graphene and IPA mixture gave how graphene particles are distributed into IPA and graphene distribution on thin solid film. During filtration, cluster of graphene particles are observed due to electrostatic force, hence few particles are not filtered through filtration because graphene particulates are larger than the size of the filter paper. The thickness of the film, conductivity and roughness are determined for PEDOT:PSS incorporation without doping and doping substrates with different vibration frequency [144]. It was investigated by adding more IPA with PEDOT:PSS solution can increase the conductivity but lowering the uniformity as well as increase the surface defects force to decrease concentration of precursor solution so film roughness will increase [145]. The mechanical strength of the film will improve due to higher conductivity. The graphene worked as bridge to modify the inner structure and surface that causes increase in the conductivity ten times more by utilization of graphene and PEDOT:PSS solution. The graphene was doped with PEDOT:PSS at lower vibration frequency and low power will increase the conductivity rather than conventional method. However, at higher frequency, the conductivity will less and quality of the film decline [146]. The X-ray diffraction (XRD) analysis established the presence of low intensity peak at 23 °C for all type of film made with PEDOT:PSS [142,147]. The peak at 26 °C confirmed the effect of conductivity of graphene and dispersion on PEDOT: PSS matrix but peak with undoped IPA result was identical. The UV–Vis transmission indicated the behavior of graphene and PEDOT:PSS film (Figure 5). The film prepared by substrate vibration-assisted spray coating with less thickness and desirable uniformity have the superior transmission compare to standard coating method. 

### 2.3. Poly(4-Butyltripheneylamine) (PTPA) and Polystyrene (PS)

**Figure 6:**
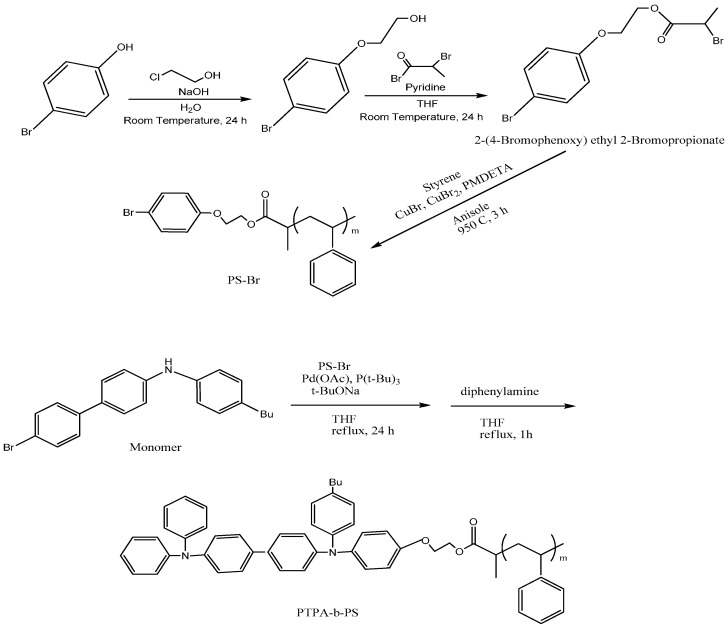
Synthesis of PTPA-*b*-PS using PS-Br as a terminal modifier via C–N coupling polymerization.

2-(4-bromophenoxy) ethanol was dissolved with pyridine and tetrahydrofuran under the influence of nitrogen at 0 °C. Initially after adding 2-bromopropionyl bromide maintained the same temperature for 30 min and continues stirred for 24 h at 25 °C. Rotary evaporator was used to evaporate the tetrahydrofuran to get residue and modified the concentration of organic layer after drying. Column chromatography was used to purify the product 2-(4-Bromophenoxy) ethyl 2-Bromopropionate [148]. The yellow liquid product, styrene, copper(I)bromide, copper(II)bromide, *N*,*N*,*N*′,*N*′,*N*″-pentamethyldiethylenetriamine (PMDETA), and anisole were added under nitrogen and continued stirring for 3 h at 95 °C. The precipitate PSBr was formed due to atom transfer radical polymerization [149] and purified using methanol. The monomer was dissolved with tetrahydrofuran, sodium tertiary butoxide and added with prepared mixture of PS-Br, palladium(II)acetate, tri-*t*-butylphosphine, tetrahydrofuran under nitrogen. After stirred at reflux temperature for 24 h, diphenylamine solution was combined with it and continues stirring. The pale greenish precipitation of poly(4-butyltriphenylamine)-*b*-polystyrene (PTTA-*b*-PS) (Figure 6) was formed and dried with acetone.

The nuclear magnetic resonance (NMR) spectra gave the evidence of presence of homopolymers. It shows the signals of PTPA backbone and PS segment represents a new signal in aliphatic, aromatic regions. The methylene units junction found in PTTA-*b*-PS and PS-Br are 3.6 and 4 ppm respectively. Therefore, it was confirmed PS successfully inserted into PTTA. The weight ratio of PS segment was in the range of 5.3 to 39%, comparing the integral ratio of meta protons of styrene ring at 6.6 ppm and methylene protons of butyl group at 2.6 ppm [148]. Differential scanning calorimetry (DSC) measured the thermal properties of PTPA-*b*-PS. The glass transition temperature of PS and PTTA are 70 and 180 °C respectively. PS content and homopolymer are very less as well as one glass transition temperature present in the PTTA segment of PTTA-*b*-PS [148].

### 2.4. Poly[2-Methoxy-5-(30,70-Dimethyloctyloxy)-1,4-Phenylenevinylene] (MDMO-PPV) and Lead (II) Sulfide (PbS)

Initially orange semi-transparent solution was prepared by dissolving MDMO-PPV in toluene and changed completely into transparent solution by addition of dimethyl sulfoxide. Then lead acetate added and dissolved in the solution. Slowly orange solution was converted to dark after mixing with thioacetamide. The solution heated in steel autoclave at 160 °C for 24 h. The red precipitates dried in ethanol and byproducts are extracted by centrifugation [150].

The energy dispersive spectroscopy on nanorods proved presence of PbS and showed main elements are Pb and S. The absorption properties are studied using UV–Vis–NIR spectroscopy. The absorption range was wide from UV to NIR demonstrates by absorption spectra of MDMO-PPV-PbS and MDMO-PPV. The MDMO-PPV attached with ligand PbS and proved by present peak from 400 to 550 nm in the spectra. Therefore, it indicates that the absorption of MDMO-PPV can be improved by incorporating PbS into the matrix. 

### 2.5. Poly (3-Hexyl Thiophene) (P3HT) and Polystyrene (PS)

**Figure 7:**
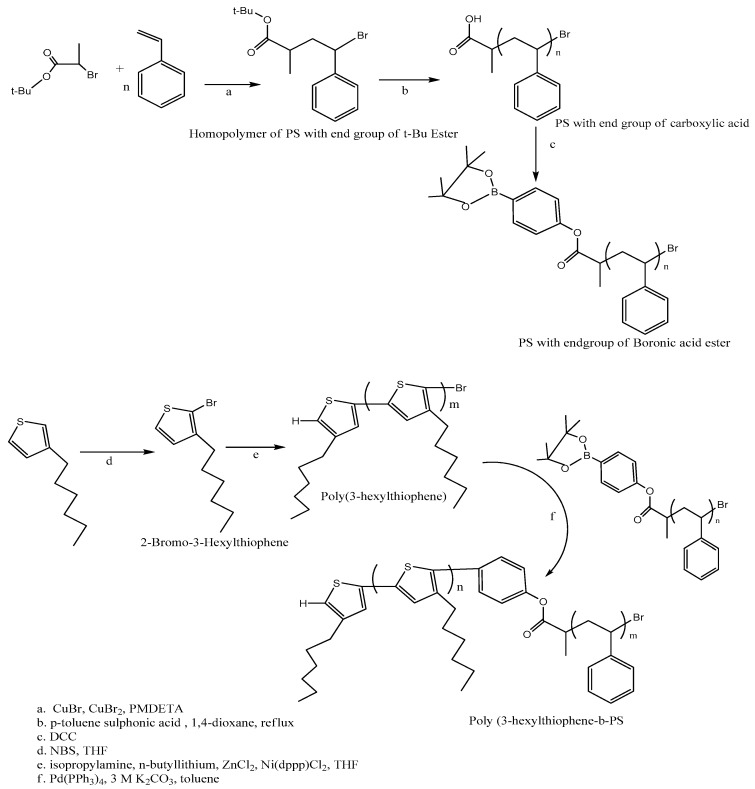
Synthesis route of diblock copolymer P3HT-*b*-PS.

Styrene, anisole, copper(I)bromide, copper(II)bromide, *N*,*N*,*N*′,*N*′,*N*″ pentamethyldiethylenetriamine and 2-bromopropionic acid are mixed and under nitrogen heated for 24 h with temperature 90 °C. The product 1 was formed by atom transfer radical polymerization [149]. The precipitate and *p*-toluenesulfonic acid were dissolved in dioxne. The mixture was refluxed under nitrogen at 95 °C for 24 h. The precipitate was combined with 4-(4,4,5,5-tetramethyl-1,3,2-dioxaborolan-2-yl)phenol, dicyclohexylcarbodiimide and *N,N*-dimethylaminopyridine with continue stirring for one day. The precipitate was separated by filtration. 3-hexylthiophene was dissolved in tetrahydrofuran. 2-bromo-3-hexylthiophene was synthesized after *N*-bromosuccinamide incorporated into the mixture and purified by column chromatography. Diisopylamine and *n*-butyllithium were transferred in a flask that contain tetrahydrofuran with temperature −78 °C. 2-bromo-3-hexylthiophene and anhydrous zinc chloride were mixed with the solution with interval of 5 min. The solution was combined with bis(diphenylphosphino) propanedichloronickel (II) (Ni(dpp)Cl_2_) with continue stirring. The mixture was diluted with methanol and used hexane, methylene chloride to extract the product by Soxhlet [151]. The copolymer P3HT-*b*-PS are synthesized by utilizing tetrakis(triphenylphosphine)palladium(0), potassium carbonate, polystyrene, poly-3-hexylthiophene and toluene. Initially mixture was refluxed and then stirred at 100 °C for 24 h. Methanol and acetone were used to wash the synthesized product (Figure 7) [152].

The nuclear magnetic resonance (NMR) studies of polystyrene with tertiary butyl ester indicated the end of CH_3_CH and C(CH_3_)_3_ group. The degree of polymerization depends on intensity of parent signal to aromatic protons. The gel permeation chromatography (GPC) provides polydispersity and average molecular weight 1.23 and 2300 respectively while thermogram of differential scanning calorimetry gave glass transition temperature 65 °C. The tertiary butyl group was eliminated from polystyrene attached carboxylic acid and confirmed by NMR as well as with IR spectrum by absence of tertiary butyl group and decrease in the doublet absorption. The purity of 2-bromo-3-hexylthiophene was determined by high performance liquid chromatography and purity can be increased by passing through column chromatography. The polydispersity and average molecular weight of poly(3-hexylthiophene) are found 8100 and 1.49 respectively with GPC and degree of polymerization by NMR [153]. The increase in the molecular weight to 10,000 confirmed the formation of copolymer P3HT-*b*-PS shown by GPC. The NMR spectrum gave the signals of aromatic regions that indicate PS segment contains proton on phenyl rings at 6.7 and 7 ppm. UV–Vis spectroscopy evaluated the aggregation state of P3HT. Thermal and solvent annealing of the films provided similar absorption spectra with maximum wavelength 560 nm [152]. The absorbance was slightly increased after solvent and thermal annealing treatments at 607 nm, established higher crystallizability with strong intermolecular interaction in P3HT. Therefore, the PS block does not disturb the surface of P3HT block. Morphology study of P3HT-*b*-PS was investigated by AFM. There is no unique structure available before annealing only flat surface. After solvent annealing, phase was clear due to separation. Therefore, PS block formed a structure on the thin film that thermodynamically stable prompt to design more indistinct dense structure of P3HT.

## 3. Solar Energy Conversion Technology

### 3.1. Energy Generation Principle

Solar cells generate energy by electron displacement, this happens when the free electrons in the surface of the panel, and the photons that come from the sun collide [107]. Solar cells are composed of a p-n junction, the first is denominated “p” and it is full of electrons, and the second is denominated “n” and lacks electrons. It is also known as “holes”. The electric field between these 2 zones make possible the movement of electrons from “p” to “n”, but not in the other direction by natural means. Following this principle, when using layers of different materials with different band gaps, efficiency tend to increase, for these reasons multi-junction cells are studied. Considering that anode “p” should not be too large, because it will block incoming sunlight and if it is too small, it will have a bad conductivity. In addition, conductive grids are implemented in the surface of the cell. Lastly, an anti-reflective coat is applied in the glass cover to protect the solar cell [107]. 

#### 3.1.1. Solar Radiation

Sunlight is composed of several different radiation specters; this varies significantly in different locations because of latitude, humidity, temperature, angle of incidence, among other reasons [154]. For the spectral irradiance of the sunlight, the performance of solar cells is assessed by two parameters; these are the average photon energy (APE) and the spectral mismatch factor (SMF). APE is the integrated radiation divided by the integrated flux density; the result is energy per photon (eV), by this parameter it can be identified if the spectrum shifts relative to red or blue enriching this type of light [155]. SMF represents the energetic gain and at the same time the loss in effective irradiance available to the solar cell, respect total experimental irradiation, compared to the theoretical spectral irradiance [156]. When SMF is greater than 1 means that solar spectral distribution is going to produce short circuit. These short-circuit currents indicate spectral losses. In addition, SMF less then 1 indicate spectral gain [157]. Other studies about the influence of radiation in the solar cells have been made in recent years [158,159].

Solar radiation supplies the necessary energy to drive the earth climate. This radiation interrelates with both the atmospheric and electromagnetic envelopes leading large solar radiation across the globe for transformation to other energy types such as the Normal daylight (380–850 nm), Silicon-based PV cells (350–1100 nm), plants (400–700 nm) and wavelength, respectively. Contemporary broadband solar radiation measuring devices include the pyranometers, complete cavity radiometers, pyrheliometers applies advanced manufacturing techniques. In designing a solar PV, identifying the constituents such as diffuse or hemispherical solar radiation is the initial stage for design evaluation. Table 2 below shows the solar radiation normally requires by solar planers and designers.

#### 3.1.2. Light Harvesting

Improving the performance of a solar cell can be achieved by decreasing the bandgap or managing the photons [111]. The first relies on the semiconductors, in as much as they can only absorb photons with higher energy than its bandgap [111]. Although photons with higher energy than the bandgap tend to excite electrons in lower energy levels, to rise above the conducting band minimum (CBM), then these release the extra energy as heat while they relax to the CBM, thus exist an optimal bandgap [160,161]. On the other hand, the transmission of light is unavoidable, but these transmitted photons are not wasted, they can be used as trough cells staked properly [162,163] or transparent solar cells [164,165]. Also photon excitation can be maximized by the process of down-conversion [166] and up-conversion [167,168].

#### 3.1.3. Efficiency of Charge Transportation and Collection

Overall efficiency of the solar cell can be increased by improving the specific efficiency of the charge transportation and collection [111]. In the charge transportation two aspects can be optimized, the first is “charge drift” and happens when charge travels under the effect of an energy field, the second is “charge diffusion” and occurs when a charge moves under a charge concentration gradient, accelerating this phenomena improves charge transportation [111]. For organic cells, the diffusion length tends to be 10 nm [169,170]. Usually, it requires to be 100 nm thick, for effective absorption [111]. This issue is solve in bulk heterojunctions with fine grains [171,172], where the grain size must be carefully chose [173,174]. Charge collection should be quick to prevent charge accumulation; which could lead to recombination at the interface [111]. This collection efficiency can be improved by the use of 1D electrode, that manifest as a charge collection highway, this has been done with nanotube arrays [175] and interlacing two 1D polymers in organic cells [176,177].

### 3.2. Electric Generation Through Organic Solar Cells

The world global population is now exciding 7 billion, which implies a greater energy demand [178]. It has been calculated that this demand could by supplied, while the amount of solar energy that impacts the surface of the earth in less than an hour [179]. Solar energy is environment friendly, because it does not pollute like the combusting fossil fuels, this could help decrease the ${\mathrm{CO}}_{2}$ concentration that in the year 2012 reached 393 ppm, which is past the safe threshold of 350 ppm [180]. At the end of the year 2014 the photovoltaic installed capacity figured less than 177 GW [181] and it is forecasted that for the year 2019 this energy will reach 498 GW [182]. Due to the light weight and lower fabrication cost organic solar cells are above inorganic Silicon solar cells [183]. In this sense, the electric generation process through solar cells depends on the type of cell and the materials implemented in its manufacture. In this section, the materials and structures organic solar cells will be described, to explain in depth the electric generation.

#### 3.2.1. Types of Solar Cells

Energy generation through a solar cell depends of several factors, in general terms they are classified by composition and structure, as the following types [179]:

1. First Generation: Single (p-n) junction mono or multi crystalline silicon solar cells, the mono crystalline solar cell has an efficiency record of 25% [184].
2. Second Generation: Thin films is currently composed of copper indium gallium selenide (CIGS) [179]. This type of cell has achieved efficiency of 20.4% on flexible polymer substrate [184,185]. Low manufacture cost and high efficiency, may lead this type of cell to have a great share in the solar cell market [74].
3. Third Generation: Organic solar cells (OSC), Dye Sensitized Solar Cells (DSSC) and multijunction cells [109]. The (OSC) and (DSSC) have the following maximums of efficiency recorded 12% [186] and 11.3% [187] respectively. On the other hand multijunction cells focus on increasing power respect cost ratio, by maximizing the solar spectrum they can capture [182].

#### 3.2.2. Organic Solar Cells

The Organic solar cells (OSC) belong to the third generation type, and are composed of organic semiconductor materials [188]. This organic materials are carbon compounds and their derivatives such as organic polymers denominated “plastics” or “synthetic rubber” [188]. These materials have properties that are attractive for the photovoltaic applications [189,190], among these are [191,192]:

1. Wide range of very cheap materials and structures.
2. High absorption coefficient.
3. Ease of processing
4. Mechanical Flexibility.
5. Non-toxic.
6. Adjustable band-gap.
7. Control over the electric conductivity.
8. They can be applied at room temperature.

Among the advantages, the following stand out:

1. Low energy payback time: The energy payback time (EPBT) is the amount of time required for the solar cell, to generate the amount of energy use in its manufacture [193]. This is a life cycle metric that achieves 1% efficiency at short-term (life time of 2 years), 10% efficiency at midterm (life time of 10 years) and 15% efficiency in long-term (life time of 20 years) [194]. In this aspect (OSC) have a better performance [194].
2. Greenhouse gas emission: The greenhouse gas (GHG) emission of the solar cell reflects the impact of this in the global climate [188]. In the current scenario, this value is higher in (OSC), that in comparison to other types of solar cells [188]. In the other hand for the long term scenario this changes drastically, where (OSC) become the lesser emmiters of (GHG), due to increase of conversion efficiency and operating lifetime [188].
3. Power conversion efficiency: To increase the efficiency a wider spectral absorption range is required, this has exceed 9% single junction [195,196,197] and 11% for tandem-junction solar cells [198]. In single-junction (OSC) the film thickness of the photoactive layer is minimized to prevent recombination losses [188]. Theoretically the power conversion efficiency can be reduced to 25.5% by minimizing the loss of non-radiative voltage in fullerene-based organic solar cell [199].

For organic solar cells there are several challenges to overcome, these are [179]:

1. Tandem architectures.
2. Plasmonics.
3. Improvement upon the short diffusion length of excitons.
4. Polymeric nano-composites including graphitic nano-structural material.
5. Donor-Acceptor interface improving the number of excitons.
6. Crystal structure improvements to increase the electrical conductivity.
7. Maximizing the number of photogenerated carriers.

Organic semiconductors can be composed of the following semiconductor materials [179]:

1. Macromolecule dyes.
2. Dendrimers.
3. Pigments.
4. Oligomers.
5. Polymers.
6. Small molecules.
7. Others.

The most famous semiconductors in current and previous researches of organic solar cells (OSC) are polymers and small molecules [179]. Small molecules Are the pigments and dyes (such as anthracene, pentane, TPP-tetrafenyl prophyrins, Alq3-8-hydroxyquinolyne aluminum, chlorophyll, perylene pigment, C60–Fulerene among others) [180]. Polymers represent PFO-polyflorin, PPV-polyphenylene vinyl, MEH-PPV-polymetroxy ethyl-hexyloxy phenylene vinyl to name a few [179]. Polymers can be classified as Low band-gap polymers, Medium band-gap polymers, and Wide band-gap polymers. In recent years, a great interest in the use of low band-gap polymers as donors to realize high-efficiency polymer solar cells has emerged. Among the low band-gap polymers, the PTB7-Th (poly{4,8-bis[5-(2-ethylhexyl)thiophen-2-yl]benzo[1,2-b;4,5-b′]dithiophene-2,6-diyl-alt-[4-(2-ethylhexyl)-3-fluorothieno[3,4-b]thiophene]-2-carboxylate-2-6-diyl}), which is a derivative of PTB7 (poly{[4,8-bis(2-ethylhexyloxy)benzo(1,2-b:4,5-b′)dithiophene]-2,6-diyl-alt-[4-(2-ethylhexyl)-3-fluorothieno[3,4-b]thiophene]-2-carboxylate-2-6-diyl}), has shown huge potential in recent studies thanks to the possibility of exploiting deposition protocols (technique, solvent, concentration) from the PTB7, and the fact that it shows a power conversion efficiency (PCE) exceeding 10% [200].

**Figure 8:**
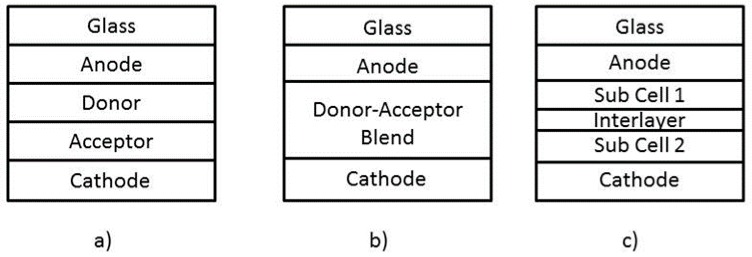
Different types of multiple layer solar cells: (**a**) Bilayer heterojunction, (**b**) Bulk heterojunction and (**c**) Tandem heterojunction [179].

Organic solar cells generate electricity in different forms depending of the solar cell structure, this are [179]:

1. Single layer: A solar cell composed of a single active material [179], usually requires a Schottky barrier in one of its contacts to allow the separations of photo excitations at the barrier field.
2. Multiple layer or Hetero junction: A solar cell composed of multiple layers with different materials, some of this materials have low ionization potential (IP/LUMO) and act as Donors, while some of this materials have a high electron affinity (EA/HUMO) and act as Acceptors [201,202]. This can be classified according to Figure 8:
  (a) Bilayer heterojunction (Planar heterojunction): Made of two layers, donor and acceptor between two electrodes [179].
  (b) Bulk heterojunction (Dispersed heterojunction): Composed of a blend between donor and acceptor, provides an easier exciton diffusion and dissociation [203]. This type of solar cell is the most investigated nowadays [204,205].
  (c) Tandem heterojunction: This type of solar cell has a two sub cells that complement the solar spectrum absorption, this sub cells are separated by an interlayer, which collects the holes and electrons generated by the cells [179]. Each sub cell is created to cover a specific region of the solar spectrum [203]. A great disadvantage for the single junction solar cells is photo-voltage loss, due the thermalization of hot carriers [206]. The organic tandem solar cell does not have these limitations, because of the Van de Waals bonding’s, this leads to a low cost and high efficiency [179].

### 3.3. Working Principle of Organic Solar Cells

Solar cells generate energy by electron displacement, this happens when in a multiple layer cell, an electron in the highest occupied molecular orbital (HOMO) absorbs a photon, and by consequence is excited in the lowest unoccupied molecular orbital (LUMO), this generates a bound electron-hole pair called exciton [207]. Extra photon energy dissipates as heat; because of this, an efficient solar cell works in a wide solar spectrum, to create the greater amount of excitons [207]. Excitons are generated due a low dielectric constant of the organic materials [208].

Exciton diffuse in the donor-accepter interface (D-A) [207]. If the exciton is inside the diffusion range of the organic polymer [209] it can be separated in a free electron and a hole denominated charge carriers [207]. This happens when the exciton meets the electric field within the diffusion range (≤20 nm) [210]. Charge carriers are swept to their respective electrodes, because of the built-in field [207]. During the diffusion recombination process will happen leading to losses, before reaching the (D-A) interface [208]. The charge carriers diffuse to the electrodes at the opposite end of the cell, flowing by an external load generating electricity [207].

The efficiency of this process rely in three aspects, first the charge carrier mobility, second the internal electric field that swept charge carriers and finally carrier recombination rate [207]. Photo generated excitons have a small life span, this implies when they not reach the (D-A) interface they are lost because of self-recombination [207].

#### Principle of Electrical Generation through Solar Cells

**Figure 9:**
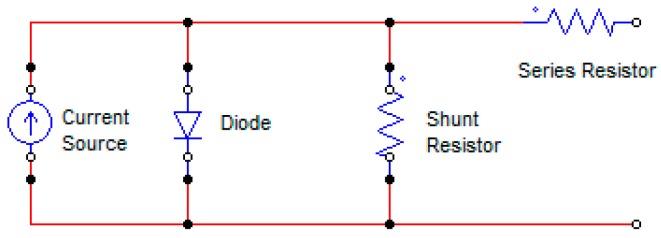
Equivalent circuit for a solar cell.

Solar cell electric generation phenomena have a circuit analog, which it allows further study. In this circuit analog the solar cell works as a diode in the dark, meaning the I-U curve goes through the origin, but in the light the solar cell this curve moves downwards [211,212]. The following circuit presented in Figure 9 can represent this.

The parameters and electric properties have been defined in several references [192].

1. Open circuit voltage $\left(U_{oc}\right)$: Is the maximum voltage across the cell, and its generated when no current is flowing through an illuminated solar cell, this happens when the voltage output terminals are open [179]. Also it can be obtained by the difference between the HOMO of the Donor and LUMO of the Acceptor [213].
  $$U_{oc}=\frac{1}{e}\left(\left|E_{HOMO}^{Donor}\right|-\left|E_{LUMO}^{Acceptor}\right|\right)-0.3$$
2. Short circuit current $\left(I_{sc}\right)$: The current flowing in an illuminated solar cell, with no external resistance connected, this is the maximum amount of current the solar cell can achieve [179].
3. Maximum power point (mpp): Is the magnitude of voltage $\left(U_{mpp}\right)$ and current $\left(I_{mpp}\right)$, which yields the maximum power in the solar cell [179].
4. Fill factor (ff): is the ratio of generated power respect the maximum power it could produce [179].
  $$ff=\frac{I_{mmp}\;U_{mmp}}{I_{sc}\;U_{oc}}$$
5. Power conversion efficiency (PCE): This magnitude reflects the electric power provided respect the total power irradiated $\left(P_{in}\right)$ to the surface of the solar cell [106].
  $$PCE=\eta =\frac{I_{mmp}\;U_{mmp}}{P_{in}}=\frac{I_{sc}\;U_{oc}\;ff}{P_{in}}$$
6. Quantum efficiency (QE): Represents the efficiency in function the incident radiation wavelength [179].

The principal parameters to improve the energy generation of the solar cell are open-circuit voltage $\left(U_{oc}\right)$, Short-circuit current $\left(I_{sc}\right)$ and Fill factor (ff) [179].

### 3.4. Mathematical Analysis of the Electric Generation Phenomena

For a further study of the electric generation in the organic solar cell, a mathematical analysis can be modeled for 1-dimension layer pseudo-bilayer device [107]. In the donor layer the Poisson’s equation can be solved for the conservation of holes and excitons, as [107]:

$$\begin{array}{c} \nabla \cdot J_{h}^{\left(d\right)}=0\\ \nabla \cdot J_{ex}^{\left(d\right)}=G_{ex}-\frac{n_{ex}}{T_{ex}}\\ -\nabla^{2}\psi^{\left(d\right)}=\frac{e}{\varepsilon_{0}\varepsilon_{\delta }}n_{h} \end{array}$$

In the acceptor layer the Poisson equation is solved for the conservation of electrons, as [107]:

$$\begin{array}{c} \nabla \cdot J_{e}^{\left(a\right)}=0\\ -\nabla^{2}\psi^{\left(a\right)}=-\frac{e}{\varepsilon_{0}\varepsilon_{\alpha }}n_{e} \end{array}$$

In the blend layer where the acceptor and donor come in contact, the Poisson’s equation can be solved as [107]:

$$\begin{array}{c} \nabla \cdot J_{e}^{\left(b\right)}=S\\ \nabla \cdot J_{h}^{\left(b\right)}=S\\ -\nabla^{2}\psi^{\left(b\right)}=\frac{e}{\varepsilon_{0}\varepsilon_{\alpha }}\left(n_{h}-n_{e}\right) \end{array}$$

with:

- $J_{h}^{\left(d\right)}$ and $J_{ex}^{\left(d\right)}$: Hole and excitons fluxes in the donor layer.
- $J_{e}^{\left(b\right)}$ and $J_{h}^{\left(b\right)}$: Electron and hole fluxes in the blend layer.
- $J_{e}^{\left(a\right)}$: Electron in the acceptor layer.
- $G_{ex}$: Exciton generation rate in the donor layer.
- $n_{e}$, $n_{ex}$ and $n_{h}$: respectively, concentration of electrons, excitons, and holes.
- $T_{ex}$: Exciton lifetime.
- $\psi^{\left(a\right)}$, $\psi^{\left(b\right)}$ and $\psi^{\left(d\right)}$: respectively, electrical potential in the acceptor, blend, and donor layer.
- e: elemental charge.
- $\varepsilon_{0}$: Permitivity of the free space.
- $\varepsilon_{\alpha }$ and $\varepsilon_{\delta }$: respectively, permittivity of the acceptor and donor.
- ε: Dielectric constant of the blend layer.
- S: Net charge generation rate.

**Figure 10:**
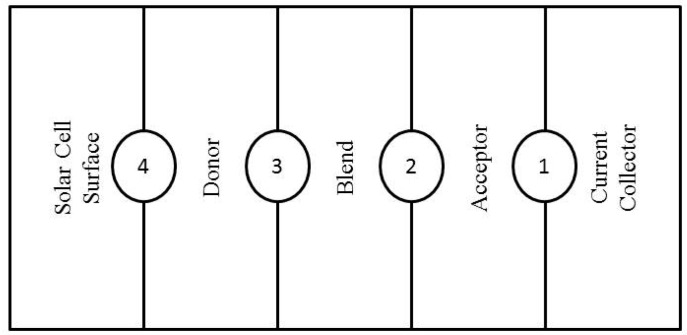
Representation of the solar cell active layers, for imposing boundary conditions.

By applying the boundary conditions in the interfaces depicted in Figure 10, the concentrations and electrical potentials can be acquired. Starting by boundary 1 between acceptor and current collector, the potential has no losses and the Boltzmann statistics delivers the concentration of electrons and holes.

$$\psi =0,\;n_{e}=N_{cv},\;n_{h}=N_{cv}\;\exp\left(\frac{-e\;U_{b}}{k_{B}T}\right)$$

with

- $N_{cv}$: Effective density of states for electrons and holes.
- $U_{b}$: Built-in voltage of the cell.
- $k_{B}$: Boltzman constant.
- T: Temperature.

In the boundary 2 between blend and acceptor the holes are assumed to stay stuck in the blend, also the electrical potential and electron flux are assumed continuous.

$$\psi^{\left(a\right)}=\psi^{\left(b\right)},\;\left(J_{e}^{\left(a\right)}-J_{e}^{\left(b\right)}\right)\cdot e_{x}=J_{h}^{\left(b\right)}\cdot e_{x}=0$$

In the boundary 3 between blend and donor excitons dissociate, increasing the flux of electrons and holes.

$$\left(J_{h}^{\left(d\right)}-J_{h}^{\left(b\right)}\right)\cdot e_{x}=-J_{e}^{\left(b\right)}\cdot e_{x}=-P\;J_{ex}\cdot e_{x},\;\psi^{\left(b\right)}=\psi^{\left(d\right)},\;n_{ex}=0$$

with P: Dissociation probability. 

In the boundary 4 between donor and solar cell surface/current collector the potential and charge concentration is:

$$\psi =U_{a}-U_{b},\;n_{e}=N_{cv}\exp\left(\frac{-e\;U_{b}}{k_{B}T}\right),n_{ex}=0,n_{h}=N_{cv}$$

where U: Applied voltage. 

With this set of equations the bulk heterojunction can be mathematically modeled, by eliminating the donor and acceptor of the active layers [107]. Also the bilayer heterojunction can be modeled similarly by removing the blend as an active layer [107].

## 4. Application of Organic Polymer Solar Cell

Although there are some issues impeding the commercialization of polymer solar cells such as inadequate stability (under ambient or thermal conditions) [6,214,215,216] and insufficient power conversion efficiency when compared with the conventional predecessors [217], there are various promising applications of organic polymer solar cells. Considering their pros and cons, Brabec et al. [214] has predicted that the cells have potentials to be used in three major sectors; on-grid sector i.e., building integration (52%), off-grid sector i.e., rural integration (25%), telecommunication and transportation (12%), others i.e., portable consumer products (11%). Some of the potential applications are listed as follows.

### 4.1. Building Integration

**Figure 11:**
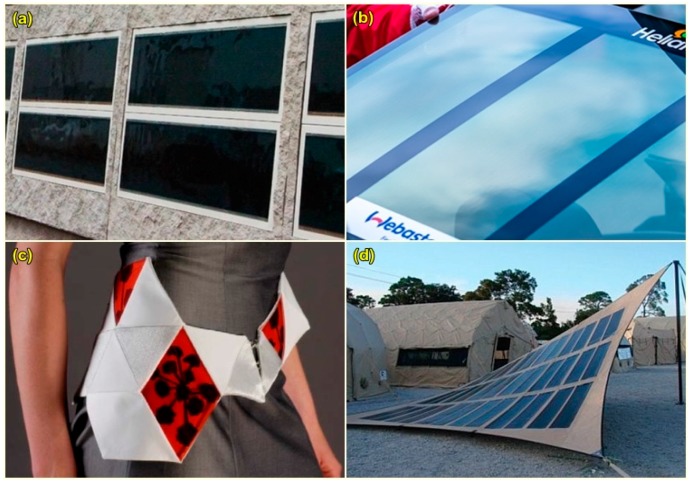
Applications of polymer solar cells; (**a**) integration on concrete façade [218]; (**b**) integration on car rooftop [218]; (**c**) integration on clothing [222]; and (**d**) integration on military tent [223].

Due to its “ultra-light, thin, highly efficient and flexible” properties [218], it is a suitable alternative to conventional solar panel design for building integration see ((Figure 11a) for example) [27,219,220]. It can be easily mounted on various building materials without the need of cooling while at the same time is aesthetically appealing [219]. This is particularly useful especially to tap into the growing global BIPV market, which is expected to reach $4.3 billion by 2021 [221]. Heliatek has carried out some real-life integration of organic polymer solar cell and some of them are listed in Table 3 below.

### 4.2. Integration on Cars

One of the applications is to integrate the cells onto vehicles. Heliatek has produced a pilot study with Webasto Automotive, where a transparent solar rooftop was integrated onto a car which acts as a powering device, as illustrated in (Figure 11b) [218]. This will reduce help the car to reduce the CO_2_ emission as part of the Original Equipment Manufacturer (OEM) requirement [218].

### 4.3. Garments, Textiles and Fabric Materials

Polymer solar cells has the capability to be integrated into fabric. Krebs et al. [222] investigated this idea by sewing the cell into the clothing see (Figure 11c). With an active area of 190 cm^2^, the cells produce a maximum power of 0.27 μW. 

DTU Energy, in collaboration with Alexandria Institute design the solar canopy and solar hammocks which were tested during SmukFest festival in 2013 [224]. The hammocks allowed the user to relax in the solar hammock while charging their mobile phone from the electricity generated from the solar hammock itself [224].

DTU and Imperial college created a yurt (a portable round tent) that powered a cinema by utilizing 60-flexible organic polymer cells [224]. The 10–12 m^2^ yurt is capable to produce 25 W of power [224]. This is a possibility of creating a portable cinema for the third world countries [224].

Konarka also produced a similar concept, and created a solar powered- tent for military used [223,225], as illustrated in Figure 11d. The tent is printed with camouflage-patterned power plastic which will reduce the possibility of being detected by the enemy, while at the same time the electricity generated can be used for charging the battery which is crucial for telecommunication-based site [225], as well as to power military equipment [226].

### 4.4. Consumer Electronics

The organic solar cell has the capabilities to be used as a “power source” to power small electronics products, such as toys, calculator, mobile phones, tablets, watches etc. This can be done by integrating the flexible cells onto the surface of those products [214,227].

## 5. Conclusions

There has been great success in both organic and inorganic solar cells used for the energy conversion process. Among them, the organic PV cell is a favorable long-term technology with low cost for manufacturing solar cells. The nanostructured oxide and polymer composite, as well as semiconductors, are developed for conversion and capable of gains energy from the sunlight. Another alternative is based on highly conductive PEDOT:PSS. It has lower sheet resistance with larger surface area and can convert more energy without substantial efficiency losses. This polymeric material will ultimately contribute towards fully printed devices and will be able to provide low-cost roll-to-roll manufacturing of solar cells. Manufacturing of eco-friendly polymer organic solar cell utilizing roll-to-roll systems obviously appears to be the future in renewable energy technologies. Recently, there has been talk of the inkjet printing technology for organic PV manufacturing because of its potential for commercial large-scale power production and low cost. As discussed by several researchers, these printing techniques may have several limitations that have to be resolved to achieve optimum result. Such limitations are dot arrangement, the likely blockage of the nozzle and limitations due its viscosity. However, the efficiency of the organic solar cells is still lower than the inorganic but recent studies show that organic solar cells are increasingly attracting attention due to their small cost, light weight and better power conversion efficiency. 

To improve the power conversion efficiency of the organic solar cells, several suggestions include diffusion of charge carriers and morphology enhancement. However, the organic solar cells are not yet commonly manufactured commercially as compared to the others and can easily be degraded with oxygen and water. Finally, for application purposes, this paper concludes that the organic solar cells have better advantages because of easier integration to many devices and systems and can be used as power source to small electronic products.

## Figures and Tables

**Table 1:** Advances and discoveries associated with solar cells over the decades.

Year	Author	Discovery
1839	Alexander-Edmond Becquerel	The first solar cell.
1873	Willoughby Smith	Selenium photoconductivity.
1876	William Grylls Adams and Richard Evans Day	Selenium harvest electrical current, when exposed to sunlight.
1893	Charles Fritts	Solar cell made of a selenium wafer.
1894	Charles Fritts	Solar cell made from selenium-coated with a thin layer of gold, this prototype had a low efficiency around 1%.
1904	Wilhelm Ludwig Franz Hallwachs	Observed photosensitivity by combining copper and cuprous oxide.
1905	Albert Einstein	Discovered the photoelectric effect that stated a good explanation of how photons are absorbed.
1916	Robert Millikan	Discovered the electron charge generated by the photoelectric effect, by measuring it.
1950	Bell Labs	Solar cells capable of energizing electric devices just by the sun radiation.
1954	Hoffman Electronics	Solar cell made of cadmium sulfide p-n junction that works with 6% efficiency.
1960	Hoffman Electronics	Solar cell made of cadmium sulfide p-n junction that works with 14% efficiency.
1962	Telstar Communications	Satellite powered by solar cells (14 W) was launch.
1972	David Carlson and Cristopher Wronski, in RCA Laboratories	The first amorphous silicon photovoltaic cells that works with 1.1% efficiency.
1980	The University of Delaware	Solar cell made of copper sulfide and cadmium sulfide thin film, which worked with greater efficiency than 10%.
1981	Paul Macready	An aircraft was made with 1600 solar cells in their wings generating 3 kW of power, flew from France to England.
1992	University of South Florida	Photovoltaic cell with efficiency of 15.9%.
1994	National Renewable Energy Laboratory	Solar cell was created achieving over 30% efficiency; this solar cell was made from gallium indium phosphide and gallium arsenide.
1999	National Renewable Energy Laboratory	Solar cell with 32.3% was developed.
2007	University of Delaware	Solar cell efficiency of 42.8%, making a world record.

**Table 2:** Data format requested by solar designers [125].

Type of Solar Data	Resolution	Application
Hemispherical, vertical surface, cardinal directions	Seasonal/daily	Glazing, building energy balance
Illuminance, vertical surfaces, cardinal directions	Seasonal/daily	Day lighting
Hemispherical tilt	Monthly/annual	Fixed flat plate
Hemispherical tracking	Monthly/annual	Tracking flat plate
Direct normal (beam)	Monthly/annual	Focusing/concentrating system
Sunshape (disk + circumsolar) variation	Varies	Concentrating tracking collector
Monthly mean daily total	Monthly/daily	Economics, design specification
Monthly mean	Monthly	Economics, design specification
Daily profiles	Hourly	System simulation, design, rating
8760 hourly data for year, hemispherical and/or direct	Hourly	System simulation, design, rating
Hourly time series 10–30-year hourly power	Hourly	Performance and economics, system lifetime
High-time resolution time series daily profiles power	Sub-hourly	Performance and economics, system lifetime

**Table 3:** Building integration of Organic polymer solar cells by Heliatek. Adapted from [218].

Year	Location	Collaborator	Building Parts/Material	Capacity
2014	Heliatek’s Dresden headquarters, Germany	AGC Glass Europe	Glass for building facade	1 kWp
2014	PuDong, Shanghai		Concrete facade	0.64 kWp
2014	Berlin, Germany	PARANET Germany	PVC-based membrane air dome	1.4 kWp
2015	Reckli Herne, Germany		Concrete facade	1 kWp
2015	vTrium Energy, Singapore		Glass and on metal	10 kWp
2016	Africa	Kandil Steel	Steel facade panels	
2016	Bergheim-Paffendorf, Germany		Profiled steel facade panels	5.4 kWp
2017	ENGIE	AGC and SVK	Fiber cement elements and onto glass	2.3 kWp
